# Estimation of cumulative number of post-treatment Lyme disease cases in the US, 2016 and 2020

**DOI:** 10.1186/s12889-019-6681-9

**Published:** 2019-04-24

**Authors:** Allison DeLong, Mayla Hsu, Harriet Kotsoris

**Affiliations:** 1 Center for Statistical Sciences, School of Public Health, Brown University, Providence, RI 02912 USA; 2 Global Lyme Alliance, Stamford, CT USA; 3 Greenwich, CT USA

## Abstract

**Background:**

Lyme disease (LD) is an infectious multi-system illness caused by the bacterial genus *Borrelia* and spread by bites of infected ticks. Although most patients are successfully treated by timely antibiotic therapy, it is broadly accepted that a sizeable number of patients experience treatment failure and continue to suffer long-term, debilitating symptoms, including pain, fatigue, cognitive dysfunction and other symptoms. This is known as post-treatment LD (PTLD), for which diagnosis is not standardized and treatment remains controversial. The prevalence and societal burden of PTLD is unknown.

**Methods:**

In an effort to help characterize the LD landscape, we estimated the number of PTLD cases in the US in 2016 and 2020 using Monte-Carlo simulation techniques, publically-available demographic datasets, uncertainty in the inputs and realistic assumptions about incidence and treatment failure rates.

**Results:**

Depending on the input assumptions, PTLD prevalence estimates for 2016 ranged from 69,011 persons (95% CI 51,796 to 89,312) to 1,523,869 (CI 1,268,634 to 1,809,416). Prevalence in 2020 is predicted to be higher than 2016, and may be as high as 1,944,189 (CI 1,619,988 to 2,304,147) cases.

**Conclusions:**

The cumulative prevalence of PLTD in the United States is estimated to be high and continues to increase. These findings will be of interest to epidemiologists and health economists studying disease burden in the US and elsewhere, and justify funding to study PTLD diagnosis and treatment.

## Background

Lyme disease (LD) is an emerging multi-system infectious illness, caused by bacteria of the *Borrelia* genus, and is transmitted by bites of black-legged *Ixodes* ticks [1]. The disease is the most frequent vector-borne illness in the United States, and is increasingly reported in Europe and Asia [2]. Researchers at the United States Centers for Disease Control and Prevention (CDC) have recently calculated annual US incidence at over 329,000 cases; however, true incidence may be higher [3, 4]. Early LD is a flu-like illness with or without the pathognomonic erythema migrans rash within thirty days of the bite. Some patients experience early neurological (facial palsy, meningitis) and cardiac (heart block, arrhythmias) manifestations.

Recent studies have shown that despite timely and standard antibiotic treatment of acute LD, treatment failure results in the chronic condition known as Post-Treatment Lyme Disease, or PTLD [5, 6]. PTLD is characterized by incapacitating fatigue, pain and neurocognitive dysfunction that persist for more than 6 months [5, 7]. Symptoms can be intermittent or constant, and are often subjective and varied in nature. There is no single case definition for PTLD, and its diagnosis is often made based on exclusion of other conditions, such as tick-borne co-infections [8]. However, LD and its sequelae are responsible for significant numbers of school and work absences, and are estimated to cost more than $1 billion per year for healthcare in the US [9, 10]. Moreover, the precise societal burden of PTLD specifically, both in real time and in projected amounts, has never been adequately quantified. Without such information, it is impossible to effectively dedicate healthcare resources, education and research efforts. As a critical first step in addressing this absence, we developed a statistical framework to estimate the prevalence of PTLD in the US by using Monte-Carlo simulation techniques and applying it under six settings representing various assumptions about the course of the US Lyme disease epidemic. The settings make use of available published data and demonstrate the wide range of measures provided in the literature. As a means of estimating the cumulative US cases, we chose two specific index dates on which to focus: 2016 and 2020.

## Methods

To estimate cumulative cases, we used inputs on Lyme disease incidence and treatment failure rates commonly reported in the peer-reviewed literature. We base our simulations on the technique presented by Crouch et al. [11] and utilize the probability distribution function from classical statistics that most closely represents the type of data used in each step of the simulation, i.e. the binomial distribution for “yes/no” data and the negative binomial distribution for overdispersed count data. We also use a simple deterministic approach as a check for the simulations. The six settings represent three scenarios for LD incidence and two treatment failure rates within each scenario. While any one of the settings may currently be considered more realistic, more research is being conducted that may change our understanding. At that time, an improved framework could be developed that incorporates all uncertainty into one simulation.

### Input data sources

#### Duration of epidemic

The first cases of Lyme disease in the United States were diagnosed in the late 1970s in the state of Connecticut [12]. LD has emerged since then, with 96% of diagnosed and reported cases found in 14 states, according to the US CDC.

#### Yearly incidence of new Lyme infections

Based on direct surveillance reporting to state health departments, there have been approximately 30,000 confirmed cases per year in the United States. However, two recent publications by US CDC researchers suggest that the actual number of new infections is much higher. Hinckley et al. (2014) estimated about 288,000 new infections in 2008 (range 240,000-444,000) based on surveys of seven national commercial labs that performed Lyme disease testing. Due to insensitivity in the diagnostic tests currently used by mainstream medical authorities, incidence estimates based solely on these tests are likely to significantly undercount the numbers of infected. Nelson et al. (2015) used data from a health insurance claims database to estimate there have been approximately 329,000 incident Lyme diagnoses per year during 2005–2010 (range 296,000-376,000). These publications show that reliance on surveillance, reported by local and regional public health authorities results in significant under-reporting.

#### Age and gender of newly infected individuals

The US CDC has estimated the age and gender distribution of new Lyme disease diagnoses in the United States. The distribution is available online at http://www.cdc.gov/lyme/stats/graphs.html (accessed June 6, 2016). This distribution shows two peaks, one in childhood (ages 5–9) that is higher among boys, and one at ages 45–55 with similar distribution by gender.

#### Treatment failure

Recent studies have shown that treatment failure rates may range from 10 to 20% [13–15]. Given the variability of treatment failure due to regional, geographical differences, socioeconomic factors, co-morbidities, treatment delays, and non-standardized treatment protocols, we chose to encompass both extremes of this range, basing our estimations on either 10 or 20%.

#### Survival

Since LD is rarely reported as a cause of death [16], we assume death rates for those with PTLD are identical to those of the general US population. Death rates for the general US population are available online at the US CDC website (accessed June 6, 2016) by gender, year (1980–2014) and 10-year age groups (except for age 0–4) [17, 18].

### Distributional assumptions for simulation

#### Incidence of Lyme disease infections

Mean: The simulations were set up to evaluate three arguably plausible assumptions about disease incidence, since the exact growth rate of the US LD epidemic is not known. All scenarios assumed 0 cases prior to 1981 and allowed linear growth between years for which estimates are available. *Scenario A* represents LD cases captured for surveillance purposes and assumed linear growth from 0 cases in 1980 to 34,449 cases in 2005, and remained stable with 34,449 annual cases from 2005 onward. These values derive from surveillance data published by the US CDC [19]. *Scenario B* similarly assumed 0 cases in 1980, linear growth to 329,000 cases in 2005 and then a stable 329,000 cases in years from 2005 onward. *Scenario C* is the same as *scenario B* to 2005, but allowed continued linear growth thereafter. Use of linear growth in our predictions is conservative over exponential growth; the latter is a potentially realistic option in an expanding epidemic, due to rapid growth of the vector population and tick habitats [20].

Variability: While the mean (or expected number) of new infections was presented as input, the simulations allowed the actual number to vary stochastically using the negative binomial distribution with the variance set to *mu* + *mu*^2^/*size*, where *mu* is the mean and *size* is an overdispersion parameter. This allowed uncertainty to increase with a higher expected number of new infections. The dispersion parameter was estimated by fitting overdispersed generalized linear negative binomial regression models to the number of confirmed cases in the CDC surveillance data from 1997 to 2017, using linear growth over time as the only independent variable [19]. We fit two regressions due to the assumption of Scenario A of linear growth from 1997 to 2005 with constant incidence thereafter and because the CDC employed two different reporting criteria before and after 2008. The dispersion parameter estimated from the 1997–2005 data was 112, and the dispersion parameter estimated from the 2008–2017 data was 127. To be consistent with both models, the dispersion parameter, *size*, was set to 120 in our simulations.

#### Age and gender of new infections

Mean: The mean age and gender of new infections was assumed to follow the distribution provided by the CDC and remain stable for the duration of the epidemic.

Variability: Each new infection was assigned to a 5-year age group and gender using a multinomial distribution parametrized using the distribution of the CDC data. Each new infection was randomly assigned an age in years assuming a uniform distribution. After drawing a uniform [0, 1] deviate we assigned an age as follows: if the uniform deviate was [0–.2), we placed the individual in the lowest age in the age group, if it was [0.2, 0.4), the second lowest was used, etc.

#### Progression to PTLD

Mean: We assumed rates of treatment failure to be 10% or 20%.

Variability: With a wide range of reported rates for transitioning to PTLD from the literature, our study assumed these estimates were based on a relatively small sample size of approximately 500 individuals. To model the uncertainty, we drew a probability from a beta distribution for each year with variability determined by a sample size of 500. The parameters of the beta distribution for a mean probability, *p*, were *alpha* = p*500 and *beta* = (1-*p*)*500. For a failure rate of 10%, *p* = 0.10, the mean is 10%, and the 2 standard deviation (SD) range is 7.4 to 12.8%; for a failure rate of 20%, *p* = 0.20, the mean is 20%, and the 2 SD range is 16.4 to 23.6%.

#### Survival

Mean: While nine cases of fatal Lyme carditis have been recognized in national surveillance data from 1985 to 2018 in the US [21], a review of death reports and death certificates in the US from 1999 to 2003 cited Lyme disease as a rare cause of death [16, 22]. Survival rates for patients with PTLD were, therefore, assumed to be the same as the general US population and survival rates after 2014 were assumed to be as in 2014.

Variability: Survival to the next year was simulated by drawing a binomial random variable for each case of PTLD, parameterized using the CDC survival rates. Our non-parametric survival model differs from Crouch et al., who fit a semi-parametric survival model to survival data specific to their disease population [11].

### Simulation algorithm

The simulations estimate N^th^-year prevalence as described by Crouch et al. [11]. N^th^-year prevalence is the sum of cases identified in the N-1 years prior to the index date that survived to the index date plus the new cases from the current year. Therefore, 1-year prevalence is simply the number of new PTLD patients each year, and 2-year prevalence is the number of cases from the prior year that survived to the current year plus the current year’s cohort, etc. The total number of PTLD cases at an index date of 2016 is the sum of surviving cases since 1981, or the 36-year prevalence, and the total number of cases in 2020 is the 40-year prevalence.

A total of 6 simulations were performed, one for each of the three incidence scenarios at the two PTLD progression rates (3*2 = 6). For each simulation, we performed 5000 runs and provide the median and the 2.5th and 97.5th percentiles as coverage intervals (CI) of the results [23]. All analyses were performed in R version 3.5.1.

For each run, we did the following:

Step 1. Drew the number of incident PTLD cases per year by age and gender, specifically

- Drew the number of new infections each year from 1981 to 2020
- Drew a probability of progressing to PTLD for each year from 1980 to 2020
- Using the probabilities in (b), randomly assigned each new infection as recovered or PTLD
- Randomly assigned each PTLD case a 5-year age and gender category
- Randomly assigned an age in years to each case

Step 2. Allowed the cohorts to age for the duration of the epidemic, specifically

- Determined survivors to the following year using age, gender, and year-specific survival rates
- Increased the age of survivors by one year
- Added the survivors and updated as the N + 1-year prevalence
- Repeated for the duration of the epidemic and appropriately tallied the results

Step 3. Steps 1 and 2 were repeated 5000 times and the results were stored for analysis.

### Deterministic estimate of prevalence

As a check of the validity of the simulation, the expected prevalence of PTLD cases was also estimated using a deterministic approach. For each year of the epidemic, new infections were expected to follow scenario A, B or C exactly, have an age and gender distribution precisely equal to that presented by the CDC report, exactly 10 or 20% of new cases transitioned to PTLD, and death occurred at 80 years of age. The deterministic values should have approximated the mean from the simulations, but did not have associated variability.

## Results

The 2016 and 2020 PTLD prevalence estimates varied for the three incidence scenarios and two treatment failure rates (Table 1). The 2016 estimates were highly variable and ranged from a low of 69,011 persons (95% CI 51,796 to 89,312) with *Scenario A* and failure rate of 10%, to 1,523,869 (95% CI 1,268,634 to 1,809,416) with *Scenario C* and failure rate of 20%. Regardless of incidence scenario or failure rate, all results indicated an increase in the prevalence of PTLD from 2016 to 2020, with 18, 18 and 28% increases over four years under *Scenarios A, B* and *C*, respectively. *Scenario C*, with the highest 2016 prevalence estimates, also showed the largest relative growth over the following four years. *Scenario C* with a failure rate of 20% predicted a prevalence of about 2 million cases of PTLD in 2020 (1,944,189; CI: 1,619,988 to 2,304,147). Unsurprisingly, doubling the failure rate from 10 to 20% essentially doubled the expected prevalence of PTLD.

**Table 1:** US prevalence of PTLD for 3 scenarios for incidence and 2 failure rates based on a deterministic estimation and simulation. For the simulation, results are presented as the median and the 2.5th and 97.5th percentiles as coverage intervals (CI) over 5000 runs

Incidence Scenario	Failure Rate	2016		2020	
		Deterministic	Simulation	Deterministic	Simulation
A					
	10%	68,603	69,011 (51,796 - 89,312)	81,713	81,509 (61,141 - 105,591)
	20%	137,207	138,540 (114,456 - 164,408)	163,426	163,705 (135,095 - 193,979)
B					
	10%	668,303	671,876 (511,989 - 866,523)	792,572	790,411 (601,992 - 1,017,496)
	20%	1,336,607	1,351,180 (1,126,160 - 1,608,309)	1,585,145	1,590,259 (1,323,334 - 1,893,234)
C					
	10%	754,468	758,776 (575,431 - 980,601)	969,020	967,822 (732,074 - 1,249,030)
	20%	1,508,937	1,523,869 (1,268,634 - 1,809,416)	1,938,041	1,944,189 (1,619,988 - 2,304,147)

**Fig. 1:**
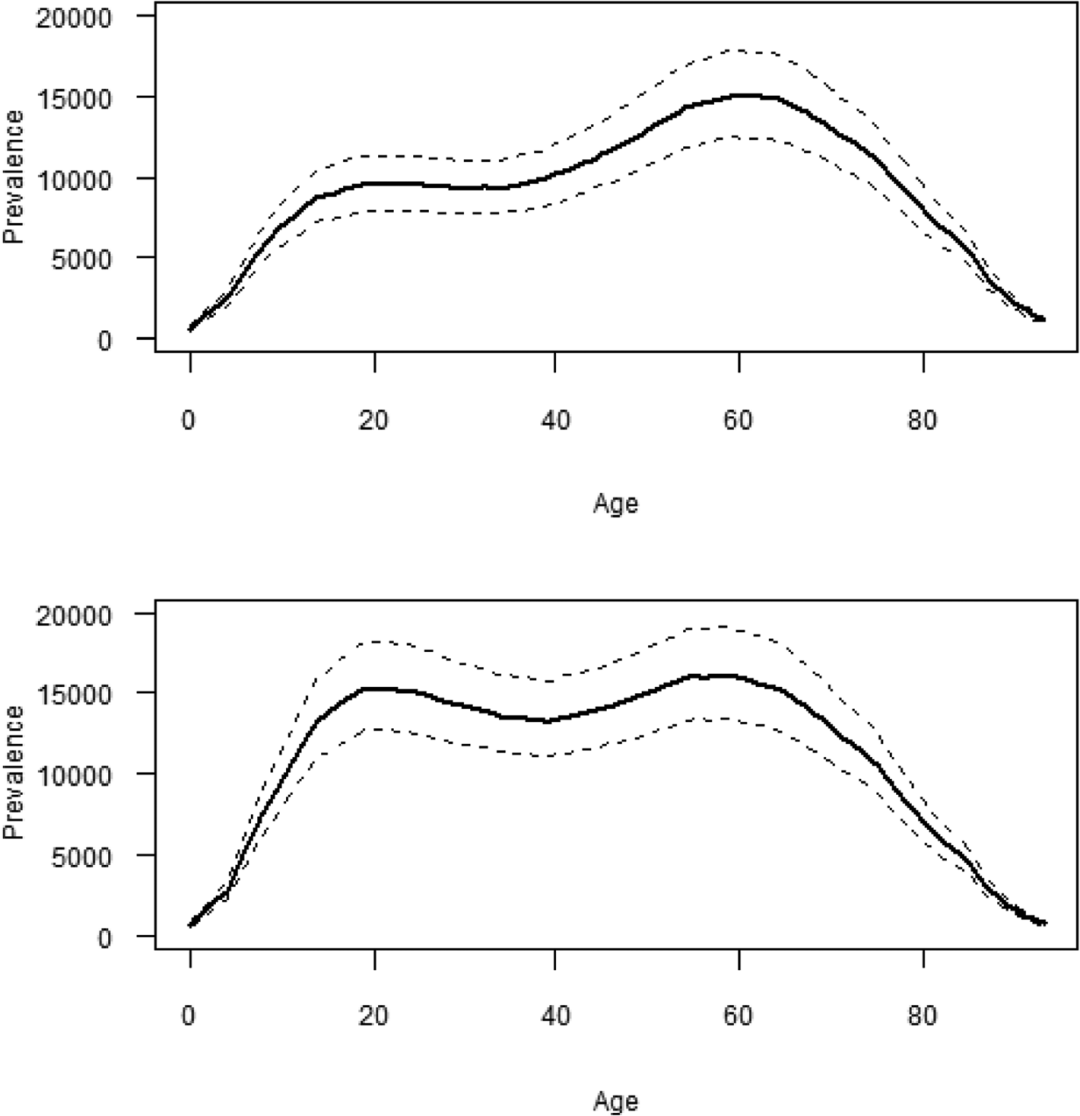
Prevalence of PTLD in 2020, by age for females (upper plot) and males (lower plot). Data are for Scenario C, with failure rate of 20%. Solid line represents the median and the dashed lines represent 95% coverage intervals

The expected number of new PTLD cases per year resulting from new infections that year is the product of the expected number of new Lyme infections and the treatment failure rate; as a result, our findings were sensitive to these input values. However, while the prevalence of PTLD varies by age and gender (Fig. 1), the relative distribution by age and gender was similar across all scenarios (not shown). In 2020 and among females, the highest prevalence PTLD was predicted among those aged 50 to 70 years; and among males, the prevalence was relatively constant among those aged 15 to 65, with peaks of higher prevalence among those aged 15 to 25 and 50 to 65.

## Discussion

Although antibiotic therapy cures most LD patients, a significant proportion of patients continue to suffer persisting symptoms that can derail normal life. These include chronic pain, neurological sequelae such as cognitive dysfunction, refractory arthritis and debilitating fatigue. Whether the persistence of symptoms is attributable to LD is the subject of impassioned debate [6, 15, 24, 25]. Due to the subjective nature of many symptoms and the absence of a diagnostic biomarker, the identification of post-treatment LD is often based on clinical diagnosis, and treatment is undefined and challenging. Diagnosing PTLD with objective, quantifiable biomarkers [26–28] is complicated and most likely involves some degree of individual variability [29]. Despite the variation in symptoms, it appears that risk factors for PTLD include delayed diagnosis and treatment as well as more symptoms and increased severity of acute LD [30–32].

The mechanisms underlying PTLD are not understood. Direct evidence of persistent pathogen replication by culturing *B. burgdorferi* from patients experiencing long-term illness is difficult. However, in the nonhuman primate model, morphologically intact spirochetes have been observed in the heart, brains, and adjacent to peripheral nerve a year after infection, with *Borrelia* antigen staining in antibiotic-treated animals [33]. Several months after infection, *Borrelia* spirochetes have been detected in antibiotic-treated mice via xenodiagnoses [34]. However, studies evaluating long-term, or repeated, antibiotic treatment of PTLD patients have not shown sustained improvement [35–38], although there is evidence some subgroups may benefit from retreatment [39]. Overall, consistent evidence for continued bacterial replication in PTLD patients is still lacking, and placebo-controlled antibiotic trials are complicated by the heterogeneity and non-quantifiable nature of symptoms in these patients. It is possible that with the development of more advanced diagnostic technology, direct detection of bacterial replication as a measurable endpoint may eventually be possible.

In addition, or alternatively, it is possible that persisting symptoms may be caused by dysregulation of the immune response to *Borrelia* infection. LD patients with continued symptoms have elevated inflammatory markers or cytokines in blood compared to those who have recovered [26–28, 40, 41], and transcription of genes involved in the response to infectious microbes may differ quantitatively and qualitatively in PTLD vs. cured patients [42].

Confounding the understanding of LD pathogenesis is the absence of a reliable diagnostic test, which causes imprecision in determining LD incidence. A two-stage blood test is recommended by current CDC protocols, and includes an enzyme immunoassay followed by a Western blot to detect antibodies against *B. burgdorferi*. Its inaccuracy, due to low sensitivity in early infection and inability to capture bacterial strain variation, is overall a dismal 50–60% [43–45]. National surveillance of LD, based on reporting from local and regional health authorities, is thus likely to be erroneous. Following antibiotic therapy, some patients may remain serologically positive, suggesting that the presence of antibodies is variable and uninformative [43]. Adding to the uncertainty is the lack of suitable biomarkers to indicate treatment endpoint and/or to suggest a transition to PTLD.

Despite the limitations in accurate diagnosis, two studies carried out by the CDC attempted to more accurately determine the incidence of LD, calculating 329,000 cases per year [3, 4]. Since the simulation models presented here are based on the expected number of new LD cases each year, this value is critical, and we incorporated the most accurate estimates known to date. We have coupled this information with data on incidence by age and gender, US life expectancy and death rates, and have constructed a simulation modeling framework to estimate the number, along with coverage intervals, of patients who continue to suffer PTLD. Our estimations for numbers of PTLD cases are between 69,011 in 2016, and ~ 2 million by 2020, suggesting a substantial number of patients living with significant health challenges.

One caveat to our models is that although LD is an expanding epidemic [46], its growth rate is not well defined. Thus, which incidence scenario is most realistic is difficult to determine. Scenario A, based on linear growth in the numbers of LD cases until 2005, followed by static low numbers thereafter, seems less plausible than the other two scenarios that include steady, sustained growth. Scenario B proposes growth until 2005, followed by a constant number of LD cases per year. This latter incidence is based on CDC estimates of 329,000 cases per year, rather than surveillance data. Scenario C is based on linear LD incidence growth from 1980 onwards.

We acknowledge and incorporate uncertainty in disease incidence and treatment failure rates by simulating six settings, each providing an estimate of prevalence under the assumption that the setting is correct. As a result, estimates of uncertainty come from the probability distribution function generating the type of data used as input, and do not incorporate uncertainty about the mechanism generating the data (i.e. the setting). The uncertainty in the data-generating mechanism is shown by examining the differences in the results across all six settings.

An alternate approach could have been to incorporate all uncertainty within one scenario, perhaps weighing each scenario based on some knowledge base. The result would be essentially a weighted average of the results presented here with very wide 95% coverage intervals. Alternatively, this question could be cast in a Bayesian framework and could incorporate expert opinion as priors. Unfortunately, as of now, the weights and priors are unknown. An improved diagnostic test, with national surveillance, and research into treatment failure rates will likely provide more precise information to indicate which setting optimally fits the dynamics of the epidemic.

Nevertheless, our findings suggest that there are large numbers of patients living with LD-related chronic illness. Due to the lack of a single case definition or single, shared phenotype for PTLD, private and public health insurance does not include the costs of caring for these individuals, who must personally pay for these expenses. Even during acute infection, LD patients are estimated to cost approximately $3000 per person annually in medical billing [9]. During long-term illness, costs are likely to be much more. A Dutch study estimated more than 1.7 disability-adjusted life years lost per patient, due to persisting Lyme borreliosis, suggesting a significant economic and societal burden [10]. Here, our estimations indicate many chronically ill US patients in present time as well as into the future. Their large numbers, as well as the economic and personal consequences they suffer, warrant further evidence-based, rigorous research to determine the causes of treatment failures, to predict which LD patients are at risk, and to design more effective therapies. Additionally, further studies are urgently needed in the LD field, to improve diagnostic tests, increase medical and public awareness, and to accurately define the numbers of infected and chronically ill.

## Conclusions

Using statistical simulation techniques, we have estimated that the cumulative prevalence of PTLD in the US is high and substantially greater than the yearly incidence. We found that prevalence in 2020 is projected to be higher than 2016, and may be as high as 1,944,189 (CI: 1,619,988 to 2,304,147) cases. These findings are relevant to consideration of expected costs for Lyme disease treatment and the care of those with PTLD.

## Abbreviations

CDC: United States Centers for Disease Control and Prevention
LD: Lyme disease
PTLD: post-treatment Lyme disease

## References

[1] Burgdorfer W, Barbour AG, Hayes SF, Benach JL, Grunwaldt E, Davis JP (1982). Lyme disease-a tick-borne spirochetosis?. Science..

[2] CDC. Lyme Disease statistics: Centers for Disease Control and Prevention; 2016 [Available from: https://www.who.int/mediacentre/factsheets/fs387/en/index10.html.

[3] Hinckley AF, Connally NP, Meek JI, Johnson BJ, Kemperman MM, Feldman KA (2014). Lyme disease testing by large commercial laboratories in the United States. Clin Infect Dis.

[4] Nelson CA, Saha S, Kugeler KJ, Delorey MJ, Shankar MB, Hinckley AF (2015). Incidence of clinician-diagnosed Lyme disease, United States, 2005-2010. Emerg Infect Dis.

[5] Aucott JN, Crowder LA, Kortte KB (2013). Development of a foundation for a case definition of post-treatment Lyme disease syndrome. Int J Infect Dis.

[6] Wormser GP, Dattwyler RJ, Shapiro ED, Halperin JJ, Steere AC, Klempner MS (2006). The clinical assessment, treatment, and prevention of Lyme disease, human granulocytic anaplasmosis, and babesiosis: clinical practice guidelines by the Infectious Diseases Society of America. Clin Infect Dis.

[7] Cairns V, Godwin J (2005). Post-Lyme borreliosis syndrome: a meta-analysis of reported symptoms. Int J Epidemiol.

[8] Aucott JN (2015). Posttreatment Lyme disease syndrome. Infect Dis Clin N Am.

[9] Adrion ER, Aucott J, Lemke KW, Weiner JP (2015). Health care costs, utilization and patterns of care following Lyme disease. PLoS One.

[10] van den Wijngaard CC, Hofhuis A, Harms MG, Haagsma JA, Wong A, de Wit GA (2015). The burden of Lyme borreliosis expressed in disability-adjusted life years. Eur J Pub Health.

[11] Crouch S, Smith A, Painter D, Li J, Roman E (2014). Determining disease prevalence from incidence and survival using simulation techniques. Cancer Epidemiol.

[12] Steere AC, Malawista SE, Snydman DR, Shope RE, Andiman WA, Ross MR (1977). Lyme arthritis: an epidemic of oligoarticular arthritis in children and adults in three Connecticut communities. Arthritis Rheum.

[13] Feder HM, Johnson BJ, O'Connell S, Shapiro ED, Steere AC, Wormser GP (2007). A critical appraisal of "chronic Lyme disease". N Engl J Med.

[14] Rebman AW, Bechtold KT, Yang T, Mihm EA, Soloski MJ, Novak CB (2017). The clinical, symptom, and quality-of-life characterization of a well-defined Group of Patients with posttreatment Lyme disease syndrome. Front Med (Lausanne).

[15] Marques A (2008). Chronic Lyme disease: a review. Infect Dis Clin N Am.

[16] Kugeler KJ, Griffith KS, Gould LH, Kochanek K, Delorey MJ, Biggerstaff BJ (2011). A review of death certificates listing Lyme disease as a cause of death in the United States. Clin Infect Dis.

[17] CDC. Mortality Tables: Centers for Disease Control and Prevention; [US mortality data]. Available from: https://www.cdc.gov/nchs/nvss/mortality_tables.htm.

[18] CDC. Cause of death: Centers for Disease Control and Prevention; [Available from: https://www.cdc.gov/nchs/data/statab/gm290-98.pdf.

[19] Schwartz AM, Hinckley AF, Mead PS, Hook SA, Kugeler KJ (2017). Surveillance for Lyme disease - United States, 2008-2015. MMWR Surveill Summ.

[20] Hahn, MB, Jarnevich, CS, Monaghan, AJ and Eisen, RJ. Modeling the Geographic Distribution of Ixodes scapularis and Ixodes pacificus (Acari: Ixodidae) in the Contiguous United States. J Med Entomol. 2016;53(5):1176–91. 10.1093/jme/tjw076 PMC5491370 27282813

[21] CDC. What you need to know about Lyme carditis: Centers for Disease Control and Prevention; 2018 [Available from: https://www.cdc.gov/lyme/signs_symptoms/lymecarditis.html.

[22] Forrester JD, Mead P (2014). Third-degree heart block associated with Lyme carditis: review of published cases. Clin Infect Dis.

[23] R: a language and environment for statistical computing. Vienna, Austria.: R Core team. R Foundation for statistical computing.; 2018 [Available from: https://www.r-project.org/.

[24] Lantos PM (2015). Chronic Lyme disease. Infect Dis Clin N Am.

[25] Weitzner E, McKenna D, Nowakowski J, Scavarda C, Dornbush R, Bittker S (2015). Long-term assessment of post-treatment symptoms in patients with culture-confirmed early Lyme disease. Clin Infect Dis.

[26] Aucott JN, Soloski MJ, Rebman AW, Crowder LA, Lahey LJ, Wagner CA (2016). CCL19 as a chemokine risk factor for posttreatment Lyme disease syndrome: a prospective clinical cohort study. Clin Vaccine Immunol.

[27] Strle K, Stupica D, Drouin EE, Steere AC, Strle F (2014). Elevated levels of IL-23 in a subset of patients with post-Lyme disease symptoms following erythema migrans. Clin Infect Dis.

[28] Uhde M, Ajamian M, Li X, Wormser GP, Marques A, Alaedini A (2016). Expression of C-reactive protein and serum amyloid a in early to late manifestations of Lyme disease. Clin Infect Dis.

[29] Strle K, Shin JJ, Glickstein LJ, Steere AC (2012). Association of a toll-like receptor 1 polymorphism with heightened Th1 inflammatory responses and antibiotic-refractory Lyme arthritis. Arthritis Rheum.

[30] Nowakowski J, Nadelman RB, Sell R, McKenna D, Cavaliere LF, Holmgren D (2003). Long-term follow-up of patients with culture-confirmed Lyme disease. Am J Med.

[31] Shadick NA, Phillips CB, Sangha O, Logigian EL, Kaplan RF, Wright EA (1999). Musculoskeletal and neurologic outcomes in patients with previously treated Lyme disease. Ann Intern Med.

[32] Kalish RA, Kaplan RF, Taylor E, Jones-Woodward L, Workman K, Steere AC (2001). Evaluation of study patients with Lyme disease, 10-20-year follow-up. J Infect Dis.

[33] Crossland NA, Alvarez X, Embers ME (2018). Late disseminated Lyme disease: Associated Pathology and Spirochete Persistence Posttreatment in Rhesus Macaques. Am J Pathol.

[34] Bockenstedt LK, Mao J, Hodzic E, Barthold SW, Fish D (2002). Detection of attenuated, noninfectious spirochetes in Borrelia burgdorferi-infected mice after antibiotic treatment. J Infect Dis.

[35] Berende A, ter Hofstede HJ, Vos FJ, van Middendorp H, Vogelaar ML, Tromp M (2016). Randomized trial of longer-term therapy for symptoms attributed to Lyme disease. N Engl J Med.

[36] Cameron D (2008). Severity of Lyme disease with persistent symptoms. Insights from a double-blind placebo-controlled clinical trial. Minerva Med.

[37] Krupp LB, Hyman LG, Grimson R, Coyle PK, Melville P, Ahnn S (2003). Study and treatment of post Lyme disease (STOP-LD): a randomized double masked clinical trial. Neurology..

[38] Fallon BA, Keilp JG, Corbera KM, Petkova E, Britton CB, Dwyer E (2008). A randomized, placebo-controlled trial of repeated IV antibiotic therapy for Lyme encephalopathy. Neurology..

[39] Delong AK, Blossom B, Maloney EL, Phillips SE (2012). Antibiotic retreatment of Lyme disease in patients with persistent symptoms: a biostatistical review of randomized, placebo-controlled, clinical trials. Contemp Clin Trials.

[40] Ajamian M, Cooperstock M, Wormser GP, Vernon SD, Alaedini A (2015). Anti-neural antibody response in patients with post-treatment Lyme disease symptoms versus those with myalgic encephalomyelitis/chronic fatigue syndrome. Brain Behav Immun.

[41] Jacek E, Fallon BA, Chandra A, Crow MK, Wormser GP, Alaedini A (2013). Increased IFNalpha activity and differential antibody response in patients with a history of Lyme disease and persistent cognitive deficits. J Neuroimmunol.

[42] Bouquet J, Soloski MJ, Swei A, Cheadle C, Federman S, Billaud JN (2016). Longitudinal transcriptome analysis reveals a sustained differential gene expression signature in patients treated for acute Lyme disease. MBio..

[43] Fallon BA, Pavlicova M, Coffino SW, Brenner C (2014). A comparison of Lyme disease serologic test results from 4 laboratories in patients with persistent symptoms after antibiotic treatment. Clin Infect Dis.

[44] Waddell LA, Greig J, Mascarenhas M, Harding S, Lindsay R, Ogden N (2016). The accuracy of diagnostic tests for Lyme disease in humans, a systematic review and meta-analysis of north American research. PLoS One.

[45] Wormser GP, Nowakowski J, Nadelman RB, Visintainer P, Levin A, Aguero-Rosenfeld ME (2008). Impact of clinical variables on Borrelia burgdorferi-specific antibody seropositivity in acute-phase sera from patients in North America with culture-confirmed early Lyme disease. Clin Vaccine Immunol.

[46] Kugeler KJ, Farley GM, Forrester JD, Mead PS (2015). Geographic distribution and expansion of human Lyme disease, United States. Emerg Infect Dis.

